# Biomechanical conditioning of the motor unit transitory force decrease following a reduction in stimulation rate

**DOI:** 10.1186/s13102-020-00208-6

**Published:** 2020-09-29

**Authors:** Joanna Rakoczy, Katarzyna Kryściak, Hanna Drzymała-Celichowska, Rositsa Raikova, Jan Celichowski

**Affiliations:** 1 Department of Neurobiology, Poznan University of Physical Education, 27/39 Królowej Jadwigi Street, 61-871 Poznań, Poland; 2 Institute of Biophysics and Biomedical Engineering, Bulgarian Academy of Sciences, Sofia, Bulgaria

**Keywords:** Motor unit, Unfused tetanus, Rate coding, Force regulation, Stimulation frequency

## Abstract

**Background:**

The biomechanical background of the transitory force decrease following a sudden reduction in the stimulation frequency under selected experimental conditions was studied on fast resistant motor units (MUs) of rat medial gastrocnemius in order to better understand the mechanisms of changes in force transmission.

**Methods:**

Firstly, MUs were stimulated with three-phase trains of stimuli (low–high–low frequency pattern) to identify patterns when the strongest force decrease (3–36.5%) following the middle high frequency stimulation was observed. Then, in the second part of experiments, the MUs which presented the largest force decrease in the last low-frequency phase were alternatively tested under one of five conditions to analyse the influence of biomechanical factors of the force decrease: (1) determine the influence of muscle stretch on amplitude of the force decrease, (2) determine the numbers of interpulse intervals necessary to evoke the studied phenomenon, (3) study the influence of coactivation of other MUs on the studied force decrease, (4) test the presence of the transitory force decrease at progressive changes in stimulation frequency, (5) and perform mathematical analysis of changes in twitch-shape responses to individual stimuli within a tetanus phase with the studied force decrease.

**Results:**

Results indicated that (1) the force decrease was highest when the muscle passive stretch was optimal for the MU twitch (100 mN); (2) the middle high-frequency burst of stimuli composed of at least several pulses was able to evoke the force decrease; (3) the force decrease was eliminated by a coactivation of 10% or more MUs in the examined muscle; (4) the transitory force decrease occured also at the progressive decrease in stimulation frequency; and (5) a mathematical decomposition of contractions with the transitory force decrease into twitch-shape responses to individual stimuli revealed that the force decrease in question results from the decrease of twitch forces and a shortening in contraction time whereas further force restitution is related to the prolongation of relaxation.

**Conclusions:**

High sensitivity to biomechanical conditioning indicates that the transitory force decrease is dependent on disturbances in the force transmission predominantly by collagen surrounding active muscle fibres.

## Background

The modulation of motor unit (MU) force during voluntary activity is attained by changes in the rate of motoneuronal firing [3, 21]. The unfused tetanic contractions are sums of twitch-shape responses to successive motoneuronal firings. The amplitude and time parameters of these responses are considerably variable [8]. Numerous studies concerning the sensitivity of MUs to the stimulation pattern indicated that force production is a nonlinear process. On the one hand shortening of a single interpulse interval evokes the catch effect [5, 10, 15], on the other hand its prolongation evokes the tetanic depression [7, 9]. Recently, a new physiological phenomenon of a transitory force decrease after a sudden reduction in the stimulation frequency was found [16]. The contractions of isolated MUs were evoked by three-phase trains of stimuli with a low–high–low frequency pattern, and the amplitude of the transitory force decrease during the last phase exceeded even 30% of the force level, which was reached at the end of the second low frequency stimulation. The mechanisms of this effect have not been explained as yet. A previous study suggested that the transitory force decrease probably has a biomechanical background, involving processes of the force transmission by collagen within the muscle, because the phenomenon was found for only one-half of studied MUs, most frequently fast-resistant (FR) ones [16]. These units in the studied medial gastrocnemius muscle were found to be distributed predominantly within the proximal compartment [27], corresponding to only 40% of the muscle length [26], which indicates that their force is transmitted by collagen structures in the distal compartment.

Therefore, in the present study, we investigated the importance of biomechanical factors for the transitory force decrease, analysing effects of muscle passive stretching and parallel tetanic contractions of other MUs in the same muscle. These two factors are expected to influence force transmission by intramuscular collagen. In addition, we tested whether a shortening of only one interpulse interval within the train at a stable frequency (the shortest possible middle high-frequency phase) is able to evoke the studied force decrease after a force increase and how a progressively increasing number of pulses at a high rate influence the described effect. Then, the presence of a transitory force decrease at progressive changes in the stimulation frequency was tested in the contractions evoked by trains of stimuli with sinusoidal changes in frequency because a sudden switch from higher to lower frequency in the motoneuronal firing rate is not observed during voluntary activity whereas trains of stimuli with sinusoidal changes in frequency are similar to the normal behaviour of motoneurons. Finally, to more deeply understand the studied phenomenon, a mathematical decomposition of tetanic contraction with the studied transitory force decrease into a train of twitch-shape responses to successive stimuli was performed. The decomposition method is based on modeling of individual twitch forces attributed to each stimulus by progressive subtraction of successive twitch-shape curves from the unfused tetanus curve in order to follow the changes of the twitch properties leading to the studied force decrease [23].

Better understanding of the studied phenomenon could improve actual knowledge of the role of rate coding in motor control. Especially, we hypothesized that the phenomenon influence the force at progressively decreasing stimulation frequency and a confirmation of this hypothesis is important to better understand mechanisms shaping muscle force when motoneurons are decreasing firing rate, a process observed usually in human experiments when force of contraction is decreasing before the end of muscle activity.

## Methods

### Ethical Aprroval

All procedures were made to minimize the suffering of the examined animals and were approved by the Local Ethics Committee for Experiments on Animals (number of Permission: 2/2015). Additionally, various principles of laboratory animal care (Guiding Principles for the Care and Use of Animals in the Field of Physiological Sciences, the Polish Law on the Protection of Animals, and European Union regulations) were followed. Experiments were performed on Wistar rats, which were bought from Wielkopolska Center for Advanced Technologies in Poznan, owner of an animal house designed to perform experiments in conditions of GLP (Good Laboratory Practice).

### Surgery and experimental procedures

Experiments were performed on 10 female Wistar rats (4–5 months) weighing 256.4 ± 35.1 g*.* Before the experiments, the rats were kept in cages equipped with running wheels to ensure a moderate level of their activity, in an animal room with a 12:12 light/dark cycle and controlled temperature (22 ± 2 °C) and humidity (55 ± 10%). All rats had unrestricted access to standard laboratory food and tap water throughout the study period.

During experiments, the animals were anesthetized (sodium pentobarbital; initial dose of 60 mg/kg, *i.p.* supplemented approximately with doses of 10 mg/kg/h). The depth of anaesthesia was controlled by the observation of pinna and withdrawal reflexes. After the experiments the animals were euthanized (sodium pentobarbital, 180 mg/kg).

The studied muscle and sciatic nerve were separated from surrounding tissues, while other hind limb muscles were denervated. Laminectomy was performed over the L2–L6 vertebrae. The ventral roots were cut close to the spinal cord. The animals were immobilized with steel clamps placed on the tibia, the sacral bone, and the L1 vertebra. The hind limb under study was immersed in a chamber filled with paraffin oil (temperature-regulated automatically at 37 ± 1 °C) and the spinal cord was similarly covered with the oil. The muscle was connected through the Achilles tendon to a force transducer (deflection of 100 μm per 100 mN), which measured both the isometric force and the muscle passive tension.

The functional isolation of single MUs was achieved by splitting the L5 or L4 ventral roots into thin filaments electrically stimulated with rectangular pulses (amplitude up to 0.5 V, duration of 0.1 ms). The MU action potentials were recorded with bipolar silver wire electrodes inserted into the muscle. The ‘all-or-none’ appearance of twitch and MU potentials at increasing amplitudes of stimuli indicated the activity of single MU. All data were recorded on a computer disc using a 12-bit analogue-to-digital converter (sampling rates of 1 kHz and 10 kHz for force and action potentials, respectively).

In this study, 122 MUs of rat medial gastrocnemius were investigated. Seventy of them (51 FR, 10 FF, and 9 S MUs) revealed the transitory force decrease of at least a 3% amplitude and were included into the experimental procedures described below. MUs were classified into fast (F) and slow (S) based on the presence of sag in 40 Hz contraction [4]. However, due to artefacts in the force recordings, weak studied effect, low force, and high noise-to-signal ratio (mainly in the S MUs) as well as considerable instability in force due to the fatigue (mainly in the FF MUs), only 46 FR units were analysed.

### The experimental protocol

All MUs tested during our experiments were initially stimulated according to following steps:

I. MUs classification and transitory force decrease appearance:
  1. Five pulses at 1 Hz (twitch forces and action potentials were recorded and averaged)
  2. A 500-ms train of pulses at 40 Hz (the ‘sag’ in the unfused tetanus was controlled)
  3. A 300-ms train of pulses at 150 Hz (the maximum force was determined)
  4. For FR MUs, a set of recordings of tetanic contractions evoked with a three-phase train of pulses at changing frequencies in a low–high–low frequency pattern as follows: a 500-ms train of stimuli at low frequency, a 300-ms train at high frequency, and a 500-ms train at the same low frequency as the initial train, respectively. The low frequencies were 15, 20, 25, 30, 35, and 40 Hz, while the high frequencies were 75, 90 and 150 Hz, and all possible combinations of low and high frequencies were tested, i.e., the protocol included 18 trials delivered according to increasing stimulation frequency.

Ten-second intervals between each part of the stimulation protocol were applied.

**Fig. 1:**
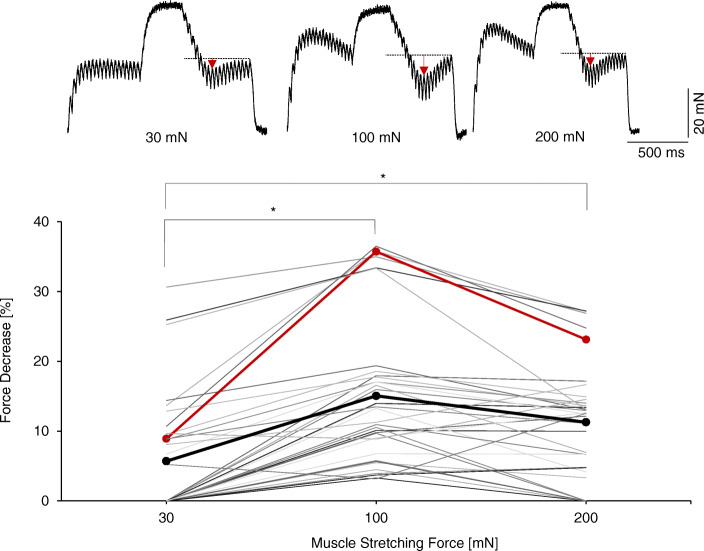
The influence of the muscle passive stretch on the amplitude of the transitory force decrease. Upper recordings: a set of sample recordings of the tetanic contractions of fast resistant motor unit stimulated with a 30–75–30 Hz pattern in a muscle stretched up to the passive forces of 30, 100, and 200 mN. The horizontal interrupted lines indicate the reference force at the end of tetanus used to calculate the amplitude of the force decrease. The red vertical arrows indicate the amplitude of the force decrease. Lower graph: amplitude of the force decrease obtained for the muscle passive stretch values of 30, 100, and 200 mN. Data for each set of recordings are connected by a line; the bold red line refers to motor unit illustrated upper. The figure presents combined data for 6 fast resistant motor units for 9 applied stimulation patterns. Note that, at 30 mN for some recordings, the transitory force decrease was not present. The black line connects the mean values at three tested levels of muscle passive stretch; level of significance of differences is indicated above. Significance of differences between groups is represented by * (*p* < 0.01)

**Fig. 2:**
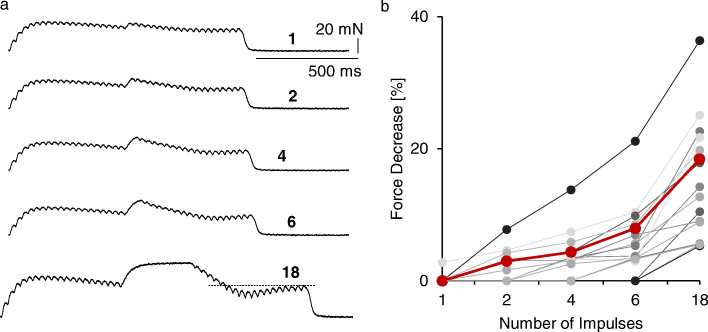
Development of the transitory force decrease at an increasing number of pulses with high-frequency stimulation. **a**, a set of sample recordings of single fast resistant contractions evoked by five trains of pulses including one, two, four, six, and 18 short interpulse intervals of 11.1 ms. Low frequency stimulation: 35 Hz; high frequency stimulation: 90 Hz. **b**, amplitude of the transitory force decrease as a function of the number of short intervals. All data for one set of recordings (obtained at low-frequency stimulations of 35 Hz and 40 Hz for all 8 examined MUs) are connected. The black line illustrates motor unit presented left. The red line presents mean values; * *p* < 0.01 in relation to one short interpulse interval

**Fig. 3:**
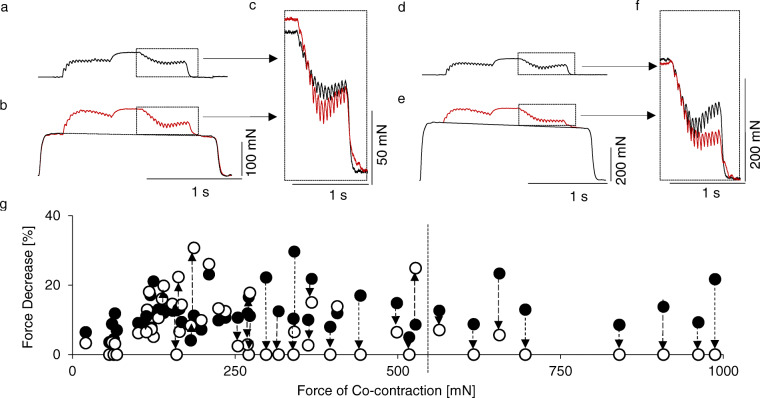
The influence of coactivity of other MUs on the transitory force decrease. **a** and **d**, the recording of two different fast resistant motor units tetanic contractions with transitory force decrease (stimulation pattern 40–90–40 Hz). **b** and **e**, recording of the same units as above evoked during parallel tetanic activity of several other motor units in the same muscle. The single motor unit unfused contraction was delayed by 250 ms in relation to the beginning of a conditioning tetanic contraction, whereas the end of this tetanic contraction was also delayed by 250 ms in relation to a single motor unit activity. **c** and **f**, the superimposed last phase of the low–high–low contractions from (**a**) and (**b**) or (**d**) and (**e**), respectively. Note that the transitory force decrease in a tetanus recorded in coactivity was increased (**c**) or reduced (**f**) depending on the force of the parallel contractions (119.0 mN in (**b**) and 353.0 mN in (**e**)). **g**, the amplitude of transitory force decrease calculated from independent recordings (black circles) or during the coactivity of other motor units (white circles), connected for the same motor unit (interrupted line with an arrow) and presented as a function of coactive tetanic force. When the force of coactive motor units exceeded 550 mN (interrupted line), the studied phenomenon disappeared, although this effect was not systematical when parallel contractions were weaker

**Fig. 4:**
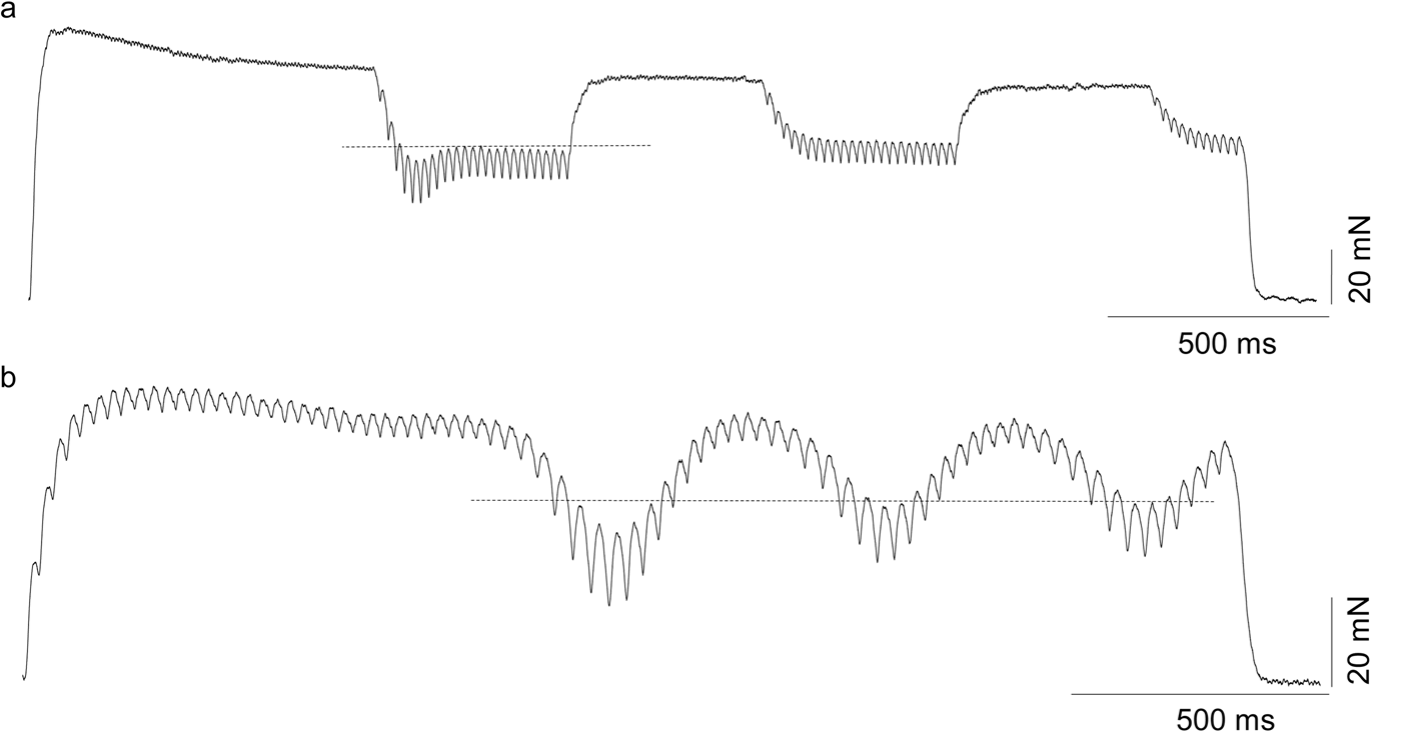
Transitory force decrease at sudden and sinusoidal changes in the stimulation frequency. **a**, sample recording of the tetanic contraction for a single fast resistant motor unit stimulated with sudden switches (at 2 Hz) in stimulation frequency (75 and 30 Hz). **b**, sample recording of the tetanic contraction for a fast resistant motor untit stimulated with sinusoidal changes (2 Hz) in stimulation frequency ranging from 9 to 16 Hz. The horizontal lines indicate the expected force at lower stimulation frequencies

**Fig. 5:**
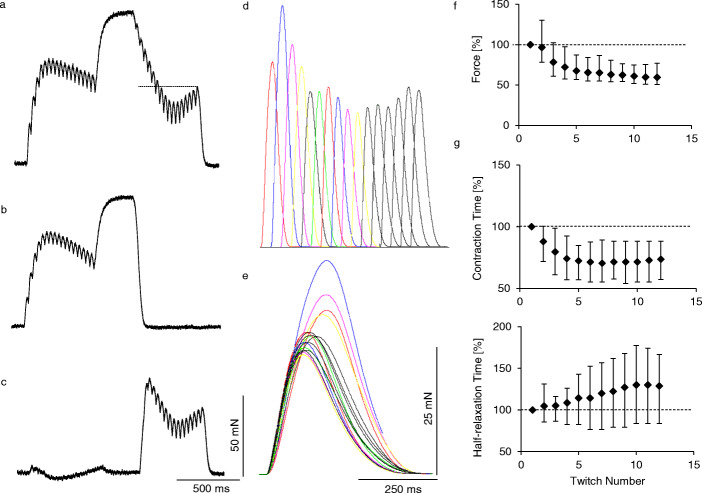
Decomposition of the tetanic contraction with the force decrease into twitch-shape responses to successive stimuli. **a**, a single fast resistant motor unit force recording with a low–high–low stimulation frequency pattern. **b**, recording of the same motor unit at a low–high frequency stimulation. **c**, mathematical difference between (**a**) and (**b**), taken for the decomposition. **d**, a train of twitch-shape responses to successive stimuli resulting from the mathematical decomposition of recording (**c**). **e**, all decomposed twitches presented superimposed with the stimulus time accepted as 0 for all twitches. The contractile parameters of successive twitches for 10 studied motor units (mean ± SD) presented in relation to the first decomposed twitch (100%): **f** – twitch force, **g** – contraction time and (**h**) – half-relaxation time (mean values and standard deviations). Due to some differences between individual motor units regarding the number of stimuli at the last low-frequency phase (minimum 12), the data are presented for 12 twitches

II. Biomechanical conditioning of the transitory force decrease:

The transitory force decrease in the third phase of the contraction was measured in relation to the force at the end of tetanus. When its amplitude exceeded 3% and the MUs were stable in force, these MUs were selected for the following additional steps of the experiment. For each of them, one of the protocols described below was applied (based on the pattern from step 4., which has evoked the strongest transitory force decrease):

1. Recordings of one to five tetanic contractions (low frequencies ranging from 25 to 35 Hz, combined with high 75 and/or 90 Hz; 35 stimulations) were repeated at the stable muscle passive stretches of 30 mN, then 100 mN, and finally 200 mN, set at least 30 s before recordings and kept constant thoughout a series of recordings (6 MUs) (Fig. 1).
2. A set of tetanic contractions with 1, 2, 4, 6 and 18 short interpulse intervals (11.1 ms) within the high-frequency phase of stimulation (i.e. in the middle part of trains of stimuli) was recorded. The number of stimuli within the low-frequency phases (35 or 40 Hz) was constant (8 MUs) (Fig. 2).
3. The selected recording was repeated during a parallel maximum tetanic contraction of a single MU or group of MUs and separately as a control before and after; additionally, this accompanying tetanic contraction was also recorded separately. The maximum tetanic contraction was evoked by 150 Hz stimulation delivered for 2000 ms (channel 1 of the stimulator), whereas the three-phase contraction (channel 2) was delayed by 250 ms and ended at 250 ms before the end of the tetanic contraction. The maximal force of coactivated MUs ranged from 20 mN up to 1000 mN (13 MUs) (Fig. 3).
4. Recordings of the force beginning at a constant phase of stimulation followed by sinusoidal changes in the stimulation frequency were done (rate of changes was 2–4 Hz, three sines; the range of changes of stimulation frequency was 7 Hz and was matched individually for each MU to evoke unfused tetanic contractions). For a comparison, instead of sinusoidal changes, sudden switches between high and low stimulation frequencies were also applied (9 MUs) (Fig. 4).
5. For 10 MUs, the decomposition of their unfused tetanus with the transitory force decrease (recordings within step 4 of the stimulation protocol) into twitch-shape responses to individual stimuli was completed according to a method described previously [23]. Changes in twitch parameters for successive decomposed responses were analysed (Fig. 5).

At the end of recordings for each MU, the fatigue test was performed (trains of 14 stimuli at 40 Hz repeated each second for 3 min) [4].

### The analysed parameters

For the single twitches, the peak force, contraction time (from the beginning of a twitch up to the peak), and half-relaxation time (from the peak up to a moment when the force decreased to 50% of peak value) were obtained. The studied units were accepted as F basing on sag in 40-Hz unfused tetanus. The fatigue index (the force generated by MU after 2 min of the fatigue test to the initial force) was calculated; for all selected MUs, the index exceeded 0.5. All data are presented as mean ± SD.

## Results

### The influence of passive muscle stretch on the amplitude of transitory force decrease

Among the three applied values of the muscle stretch (30, 100, and 200 mN), the highest effect of the transitory force decrease was observed at 100 mN (15.1 ± 10.5%), whereas, for 200 mN and 30 mN, lower amplitudes of the force decrease were noted (11.3 ± 8.2% and 5.7 ± 9.3%, respectively) (Fig. 1). Although for all selected patterns the transitory force decrease was visible at 100 mN, at 30 mN for 21 and at 200 mN for 6 out of 35 used patterns, respectively, it was abolished. The significance of differences between the compared groups (30 mN, 100 mN, 200 mN) was tested using a one-way ANOVA, post-hoc Fisher test.

### Dependence of the transitory force decrease on the number of pulses at high frequency

The effects of shortening of 1, 2, 4, 6, and 18 interpulse intervals tested for eight FR MUs at 35 and 40 Hz revealed that the studied force decrease was dependent on a number of high-frequency stimuli (Fig. 2). For all recordings, a shortening of only one interpulse interval did not evoke the transitory force decrease, but, with an increasing number of high-frequency stimuli, said force reduction progressively developed. The significance of differences between the compared groups (1, 2, 4, 6, 18 impulses) was tested using a one-way ANOVA, post-hoc Fisher test.

### The influence of the coactivity of other MUs on the transitory force decrease

The influence of a parallel maximum tetanic contraction of individual MUs or groups of units generating a large spectrum of forces (20.4–987.4 mN) on the transitory force decrease in selected MUs appeared to be dependent on this force level (Fig. 3). The separate recording with the transitory force decrease was compared to that obtained by removing the force of accompanying tetanic contraction from the common force recording (Fig. 3a vs. 3b and 3d vs. 3e), and the activity of this MU was compared to the force recorded without accompanying activity; subsequently, the differences in amplitude of the transitory force decrease in these two recordings were calculated. For each of 13 MUs, several combinations with accompanying groups of units were recorded (yielding a total of 53 sets of recordings). The amplitude of the transitory force decrease was considerably reduced or abolished when the force of coactive MU(s) exceeded 550 mN (Fig. 3g, arrows directed down). At lower forces, the studied phenomenon in some cases even increased (Fig. 3g, arrows directed up).

### The transitory force decrease during sinusoidal changes in the stimulation frequency

When the presence of a transitory force decrease was tested at progressive, sinusoidal changes in the frequency of stimulation, the force decreased to the lowest level at a low stimulation frequency within the first cycle (the mean difference in comparison to the following cycles amounted to 23.9%) (Fig. 4b). In separately recorded tetanic contractions, when the stimulation suddenly switched several times between high and low frequencies instead of presenting sinusoidal changes, the transitory force decrease was also visible at the first reduction of the frequency (Fig. 4a).

### Decomposition of the tetanic contraction with the transitory force decrease into responses to successive stimuli

To explain changes in responses to individual stimuli leading to the transitory force decrease, the decomposition of selected contractions into a series of twitch-shape responses was performed. The contraction for decomposition was obtained as a difference between the three-phase (low–high–low) and the two-phase (low–high) recordings (Fig. 5a–c. The amplitude of the transitory force decrease ranged between 5.3 and 36.4%. The analysis revealed that the force decrease resulted from a decrease of amplitudes of the decomposed twitches and a shortening in the contraction time, whereas the following force increase was an effect of a prolongation in relaxation (Fig. 5d–h).

## Discussion

The presented results indicate that a recently reported transitory force decrease [16] is not related to a decrease in the force generated by muscle fibres but rather to a deterioration in the transmission of the force to the tendon. The structures involved in this process are likely intramuscular collagen, althought it is possible that other muscle proteins as titin, the muscle fiber elastic structural protein [18] or even muscle fibres surrounding active MUs might be taken into consideration.

The transitory force decrease following a sudden reduction in stimulation frequency appeared to be dependent on several biomechanical conditions, specifically muscle passive tension (Fig. 1) and coactivation of other MUs in this muscle (Fig. 3). It is known that MU force is dependent on muscle stretches [24] as well as may be modified by the coactivity of other MUs [13]. The MUs of rat medial gastrocnemius generate the highest twitch force at a muscle passive tension of 100 mN [6], whereas the twitch duration expands with an increase of muscle passive tension. The transitory force decrease was evidently the strongest at 100 mN, which is optimal for the twitch force (Fig. 1). This observation may be related to stretch-modified properties of the collagen surrounding the contracting MU(s) or neighbouring, nonactive muscle fibres. Contracting muscle fibres pull the surrounding intramuscular collagen against the external load. Meijer [19] reported that a common system of the myofibril connections of two neighbouring muscle fibres to the same collagen in the basal lamina had an impact in force generation. The decrease in stimulation frequency evokes a sudden reduction in contractile force. The force reduction develops faster than an adaptation of passive parts of a muscle to reduced transmitted force, leading to the transitory force decrease. At a high (200 mN) or low (30 mN) applied muscle stretch, the passive structural proteins of muscle fibres have modified elastic properties, leading to a decrease in the amplitude of the studied phenomenon when compared to the optimal muscle stretch (100 mN) when the twitch force in isometric recording is the highest [6].

The transitory force decrease may be reduced by coactivation of other MUs. This observation explains why this phenomenon was not observed in similar experiments (also based on low–high–low frequency of stimulation) on human muscles stimulated via the nerve [14]. The present results show that, when the force of coactive MUs exceeded 550 mN, the transitory force decrease disappeared. In the studied muscle, the mean value of MU tetanus force amounted to 144 mN, whereas the force of the muscle stimulated via nerve was 5.25 N; the muscle consists of 52 MUs [11]. Therefore, the contraction of approximately 10 to 15% of the MUs (4–5 MUs in a studied muscle) is sufficient to abolish the studied phenomenon. It is possible that territories of two to three MUs do not overlap substantially. With increasing number of coactive MUs and overlapping of their territories, the likelihood that their muscle fibres are neighbours and transmit force by the same parts of collagen is also increased.

The MU force is sensitive to a pattern of activating stimuli; even a change in the first interpulse interval modifies the force production (see Background). Therefore, we tested whether the shortening of one interpulse interval is a sufficient enough action to evoke a transitory force decrease (Fig. 2). However, this phenomenon appeared after a longer series of pulses at a high frequency. This observation contrasts previously described effects of an initial doublet of stimuli or prolonged initial interpulse interval. The catch effect as well as tetanic depression probably depends on intracellular changes of the calcium level [1, 2, 17]. The transitory force decrease needs longer trains of high-frequency stimuli, which supports disturbances in the force transmission as a possible mechanism. This suggestion is supported by the observation that transitory force decrease occurs most frequently in FR MUs, distributed in the proximal compartment of the medial gastrocnemius [12] and covering only 40% of the muscle length [26], indicating that force must be transmitted to the tendon by long intramuscular collagen structures.

The decomposition of tetanic contractions revealed that the transitory force decrease is related predominantly to a shortening of the contraction time and a decrease in the force of decomposed twitches, followed by an increase of the half-relaxation time responsible for the final force recovery. It is worth emphasising that changes in relaxation efficiently influence the production of force during MU activity [25].

Sudden decreases or increases in the motoneuronal firing rate are not observed in voluntary activity. However, the sinusoidal changes in stimulation frequency resemble modulations in motoneuronal firing described in prior papers [20, 22]. The present results revealed that, at sinusoidal changes in stimulation frequency, the transitory force decrease influences the MU force. Therefore, the described phenomenon potentially influences the force at decreasing firings of motoneurons in vivo, in humans, leading to a force reduction occurring faster than expected, assuming a linear transmission of firing rate into the force.

## Conclusions

The transitory force decrease is a biomechanical effect most probably resulting from disturbances in force transmission from muscle fibres to tendons by adjacent collagen structures or possibly also other elastic muscle proteins. The phenomenon occurs not only during a sudden switch from high- to low-stimulation frequencies but also at progressive decreases in stimulation frequency, and therefore potentially influences the muscle force at decreasing firing rates of motoneurons during voluntary activity.

## Data Availability

The datasets used and/or analysed during the current study are available from the corresponding author on reasonable request.

## Abbreviations

F: Fast
FF: Fast fatigable
FR: Fast resistant
MU(s): Motor unit(s)
S: Slow
